# Exploration of supramolecular solvent-based microextraction for crystal violet detecting in water samples

**DOI:** 10.1016/j.heliyon.2024.e38884

**Published:** 2024-10-05

**Authors:** Najmeh Sanjarani, Mashaallah Rahmani

**Affiliations:** Department of Chemistry, Faculty of Sciences, University of Sistan and Baluchestan, Zahedan, Iran

**Keywords:** Crystal violet, Microextraction, Supramolecular solvent

## Abstract

This approach highlights the advantages of supramolecular solvents in a new microextraction model. The distinct properties and behavior of this supramolecular solvent provide enhanced extraction capabilities for detecting crystal violet (CV) in water samples. The methodical experimentation was executed to optimize the critical process parameters, providing maximum efficiency of crystal violet extraction at optimal conditions with pH set at 2.7, 186 μL of extraction solvent, extraction time of 3.5 min, and a salt amount of 3.1 % w/v, yielding the best results. Analytical data from extraction experiments under these optimal conditions demonstrated a high extraction percentage. The extraction model exhibited a linear response within the range of 10–800 ng mL^−1^ of crystal violet, with a detection limit of 2 ng mL^−1^. This model enables the measurement of CV in water samples with recovery rates exceeding 97 %, offering a straightforward and accessible approach for analysis.

## Highlights

• Green Analytical procedure based on Supramolecular Solvent for Quantification of crystal violet in water samples.
• Optimization modeling based on multivariate approaches.
• Efficiently extracting crystal violet while preserving the simplicity, sensitivity, and reliability.

## Graphical abstract

**Figure:**
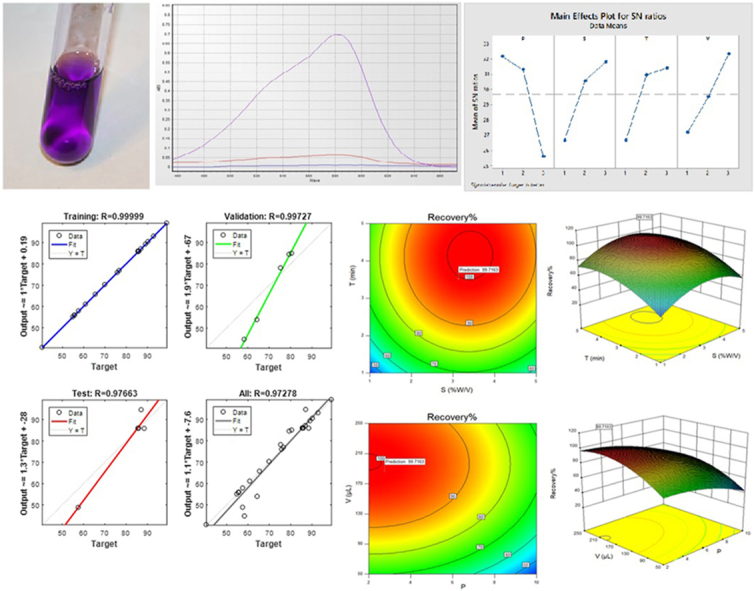

## 1 Introduction

Crystal violet is a triphenylmethane dye commonly used in histology, bacteriology, biological staining, medical, and industrial [[1], [2], [3]]. In aquaculture, crystal violet has been employed as a fungicide and antiseptic for treating fish diseases. However, its use raises concerns due to its toxicity and potential environmental impact [4,5]. Crystal violet is known to be toxic, with potential carcinogenic, mutagenic, and teratogenic effects. It can cause skin and eye irritation and has harmful effects if ingested or inhaled [[6], [7], [8], [9]]. Therefore, detecting crystal violet in water is crucial due to its persistence and potential to cause long-term environmental and health effects. Accurate measurement helps in managing pollution and ensuring water safety [[10], [11], [12]]. To enhance the sensitivity of analyte measurement, advanced analytical tools such as high-performance liquid chromatography (HPLC) coupled with mass spectrometry (MS) [13], capillary electrophoresis [14], and pre-concentration techniques like cloud point extraction (CPE) [[15], [16], [17]], solid-phase extraction (SPE) [18], solid-phase microextraction (SPME) [19], and liquid-phase microextraction methods (LPME) [[20], [21], [22], [23], [24], [25], [26], [27]] are utilized. Dispersive Liquid-Liquid Microextraction (DLLME) is characterized by its use of a small volume of organic solvent, high pre-concentration factor, simplicity, speed, cost-effectiveness, and environmental friendliness [28].

Ionic Liquids (ILs) are organic salts, typically composed of an organic cation and an organic or inorganic anion [29]. They have gained significant attention in various fields of chemistry, particularly in analytical chemistry, due to their advantageous properties. ILs exhibit properties such as high thermal stability, low vapor pressure, non-flammability, various viscosities, conductivity, and miscibility with different solvents [30]. These properties stem from the electrostatic interactions within ILs and their unique intermolecular interactions. In recent years, ILs have found widespread use as extraction agents in liquid-liquid extraction processes in analytical chemistry. They have shown promising results in extracting target analytes from complex matrices, owing to their tunable chemical and physical properties [31].

Supramolecular solvents (SUPRASs) are liquid mixtures consisting of nanostructures formed through a two-step self-assembly process involving amphiphilic compounds, occurring at either the molecular or nanometer level [32,33]. These compounds can be obtained from natural sources or synthesized chemically, with their properties being easily adjustable. The self-assembly process of SUPRAS is straightforward, typically achieved through mechanical mixing. SUPRAS offers structural flexibility and tunability, enabling customization for specific extraction applications by modifying the hydrophilic or hydrophobic groups of the amphiphilic molecules or incorporating co-surfactants. Because of these advantages, SUPRAS is extensively utilized for extracting and concentrating specific substances from various samples before analysis analysis [[34], [35], [36]].

Therefore, this study highlights the advantages of supramolecular solvents in a new microextraction approach. The unique properties and behavior of the resulting supramolecular solvent enable greater extraction capacity to monitor crystal violet in water samples. It offers benefits such as tailored selectivity, enhanced sensitivity, reduced solvent consumption, and versatility in application. Optimization of crucial microextraction parameters was achieved through multivariate techniques to enhance the efficiency of crystal violet extraction.

## 2 Experimental procedure

### 2.1 Reagents and instruments

Analytical-grade crystal violet (≥98 %), tetrahydrofuran (≥99 %), sodium chloride (≥99 %), methanol (≥99.8 %), 1-octyl-3-methylimidazolium hexafluorophosphate (≥95 %), ethanol (≥99.8 %), citric acid (≥99.5 %), acetone≥ 99.5 %), hydrochloric acid (≥37 %), phosphoric acid (≥85 %), and sodium hydroxide (≥97 %) were acquired from Merck (Darmstadt, Germany). To prepare the crystal violet stock solution, exactly 200 mg of crystal violet was measured and dissolved. This stock solution was then used to create daily working solutions for the experiment through successive dilution with distilled water. The concentration of crystal violet in the samples was determined using a UV–Vis spectrophotometer (Optizen 2120 UV Plus). The pH of the solution was measured with a pH meter (692 pH Meter, Metrohm). A centrifuge (EBA 20, Hettich) and a vortex mixer (Vortex Mixer MX-F) were used for sample preparation.

### 2.2 Samples

The proposed model for detecting crystal violet involved collecting samples of river water and fish farm reservoir water (Sistan), as well as carp fish sourced from a local supplier in Zabol, Iran. An incremental method was used in this model. First, the river water samples were clarified to remove any sediment or solid particles. For the carp fish samples, the skin and bones were removed, leaving only the tissue. Twenty grams of fish tissue were cut into pieces and mixed with 200 mL of ethanol. This mixture was treated with ultrasound waves for 30 min. Following this, 100 mL of this was separated, and 5 mL of 1 M NaOH was added to isolate the fatty acids. The resulting liquid phase was then analyzed according to the proposed model. The developed model was applied to measure crystal violet levels in river water, fish farm reservoir water, and carp fish samples.

### 2.3 Procedure

In this study, the microextraction process was conducted following the principles of the design of experiments (DoE). Sodium chloride was added to the test solution, which had a pH range of 2–10 and contained crystal violet dye at concentrations varying from 10 to 800 ng per milliliter, with salt concentrations ranging from 1 to 5 % w/v. Subsequently, 50–250 μL of extraction solvent, composed of 1-octyl-3-methylimidazolium hexafluorophosphate and tetrahydrofuran in a molar ratio of 2:1, was introduced into the test tube. This combination creates a supramolecular solvent system with unique properties that enhance the extraction capacity for crystal violet. The mixture was vortexed for 1–5 min to disperse fine droplets of the extraction solvent in the sample solution, increasing the contact surface area between the extraction solvent and the aqueous sample, and thereby facilitating analyte extraction. After vortexing, the mixture was centrifuged at 4000 rpm for 5 min to separate the extraction phase. Finally, the concentration of crystal violet in the resulting solution was measured using a UV–Vis spectrophotometer at a wavelength of 585 nm, with the results compared to a blank solution.

### 2.4 Methods

Optimizing the influential parameters in crystal violet determination from target samples is crucial. Hence, the multivariate optimization was conducted using the Design of Experiments (DoE) approach. DoE helps identify key factors, estimate their effects, detect interactions, and establish mathematical relationships between factors and responses. It systematically varies variables across experiments, considering qualitative or quantitative factors. Ultimately, DoE seeks to determine optimal factor levels for the best experimental response, helping businesses in competitive markets. In this research, we optimized crucial microextraction factors like extraction solvent volumes, sodium chloride salinity, pH, and extraction time using multivariate techniques. We utilized Taguchi [37], Response Surface Methodology (RSM) [38], and Artificial Neural Network (ANN) [39] methods to enhance a microextraction method using eco-friendly supramolecular solvent for Crystal Violet extraction. The Taguchi involves system design to identify optimal levels of parameters to determine levels that yield the best performance for the process under study. Taguchi introduced the transformation functions known as the Signal-to-Noise ratio (S/N) and Orthogonal Arrays (OAs). The optimized outcomes obtained from the Taguchi model serve as intermediate points in the RSM model to determine the crystal violet levels. Furthermore, an artificial neural network (ANN) was utilized whose input layer included critical parameters such as extraction solvent volumes, sodium chloride salinity, pH, and extraction time. The output layer represented the extraction rate to assess the neural network's performance and determine the best model for this study.

## 3 Results and discussion

### 3.1 Modeling and experimental validation

**Fig. 1:**
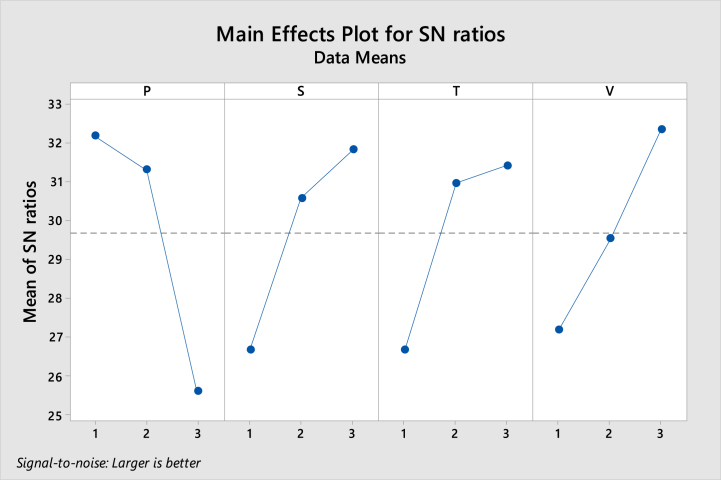
S/N ratios of Taguchi proposed for detecting crystal violet; ∗**Coded**: (1, 2, 3); **Uncoded**: **P**: pH (6, 8, 10), **S**: Salinity, sodium chloride (1, 2, 3 %W/V), **T**: Time of extraction (1, 2, 3 min), **V**: Volume of solvent (50, 100, 150 μL). (For interpretation of the references to colour in this figure legend, the reader is referred to the Web version of this article.)

Experiments were performed based on predetermined amounts for each parameter and verified the effectiveness of Taguchi model optimization using S/N ratios. Table 1 demonstrates an example of the experimental design using the Taguchi method, showcasing the effects of pH, salt concentration, extraction time, and solvent volume on the signal-to-noise ratio for different levels of these parameters. The pH was found to significantly impact the extraction process, with a range of 6–10 considered for optimization. Ultimately, pH = 6 exhibited the highest extraction efficiency. Vortexing was observed to accelerate the extraction process by dispersing the extraction solvent in the aqueous phase. Vortex times of 1, 2, and 3 min were examined, with 3 min identified as the optimal time. The addition of salt could either enhance or diminish extraction efficiency, or have no effect. Salt concentrations ranging from 1 to 3 % w/v of NaCl were investigated, with 3 % w/v chosen as the optimal concentration. Fig. 1 illustrates the signal-to-noise ratio as a function of solvent volume, showing the best response at an extraction solvent volume of 150 μL. Optimal values for the Taguchi method were determined as follows: pH = 6, extraction solvent volume V = 150 μL, vortex time T = 3 min, and salt amount S = 3 % w/v. These optimized outcomes obtained from the Taguchi model were subsequently employed as intermediate points in the RSM model to determine the crystal violet levels.

**Table 1:** The Taguchi experimental runs in detecting crystal violet.

P^a^	S	T	V	S/N ratios
1	1	1	1	23.64
1	2	2	2	34.18
1	3	3	3	38.66
2	1	2	3	32.21
2	2	3	1	31.41
2	3	1	2	30.26
3	1	3	2	24.15
3	2	1	3	26.11
3	3	2	1	26.51

a **Coded**: (1, 2, 3); **Uncoded**: **P**: pH (6, 8, 10), **S**: Salinity, sodium chloride (1, 2, 3 %W/V), **T**: Time of extraction (1, 2, 3 min), **V**: Volume of solvent (50, 100, 150 μL) for detecting crystal violet.

Table 2 displays the experimental design matrix for the central composite design coded and uncoded to achieve desirable conditions for determining the amount of crystal violet. The results varied from a low of 40.51 (run 22) to a high of 99.11 (run 12). The experiments were carefully designed to ensure comprehensive data coverage for constructing a three-dimensional surface. Influential parameters in the extraction process, including pH, salt concentration, extraction solvent volume, and vortex time, were investigated. Based on laboratory results, a second-degree model was proposed to express the relationship between crystal violet extraction percentage and the input variables.

**Table 2:** The measured and predicted runs for detecting crystal violet, with parameters entered in both coded and uncoded formats.

P (−)		S (%W/V)		T (min)		V (μL)		Recovery%	RSM predicted	ANN predicted
1	8	−1	2	−1	2	1	200	57.61	57.54	57.81
1	8	−1	2	1	4	−1	100	60.92	60.99	60.97
0	6	0	3	0	3	0	150	85.63	85.74	85.82
0	6	0	3	2	5	0	150	90.28	90.05	90.41
−1	4	−1	2	1	4	1	200	89.15	89.06	89.21
−1	4	−1	2	−1	2	1	200	75.86	75.91	75.87
0	6	2	5	0	3	0	150	80.58	80.51	84.87
−1	4	−1	2	−1	2	−1	100	58.45	58.36	44.68
−1	4	1	4	−1	2	−1	100	65.53	65.51	65.62
−1	4	1	4	1	4	−1	100	87.15	87.14	94.51
0	6	0	3	0	3	−2	50	57.59	57.59	48.77
−1	4	1	4	1	4	1	200	99.11	99.32	99.13
−1	4	−1	2	1	4	−1	100	79.41	79.48	84.39
1	8	1	4	1	4	−1	100	76.74	76.83	76.81
0	6	0	3	0	3	0	150	85.98	85.74	85.82
0	6	−2	1	0	3	0	150	54.91	54.92	54.99
2	10	0	3	0	3	0	150	64.41	64.31	53.87
1	8	1	4	−1	2	1	200	75.41	75.48	78.05
0	6	0	3	0	3	0	150	85.59	85.74	85.82
1	8	−1	2	1	4	1	200	69.97	70.13	70.18
−1	4	1	4	−1	2	1	200	85.82	85.66	85.88
1	8	−1	2	−1	2	−1	100	40.51	40.44	40.66
0	6	0	3	0	3	0	150	85.93	85.74	85.82
1	8	1	4	−1	2	−1	100	55.76	55.77	55.85
0	6	0	3	0	3	0	150	85.69	85.74	85.82
−2	2	0	3	0	3	0	150	92.93	92.98	92.94
0	6	0	3	0	3	2	250	86.94	86.88	86.97
0	6	0	3	−2	1	0	150	55.66	55.83	55.74
0	6	0	3	0	3	0	150	85.61	85.74	85.82
1	8	1	4	1	4	1	200	88.57	88.57	85.73

∗**Coded**: (−2, −1, 0, 1, 2); **Uncoded**: **P**: pH (2, 4, 6, 8, 10), **S**: Salinity, sodium chloride (1, 2, 3, 4, 5 %W/V), **T**: Time of extraction (1, 2, 3, 4, 5 min), **V**: Volume of solvent (50, 100, 150, 200, 250 μL).

**Fig. 2:**
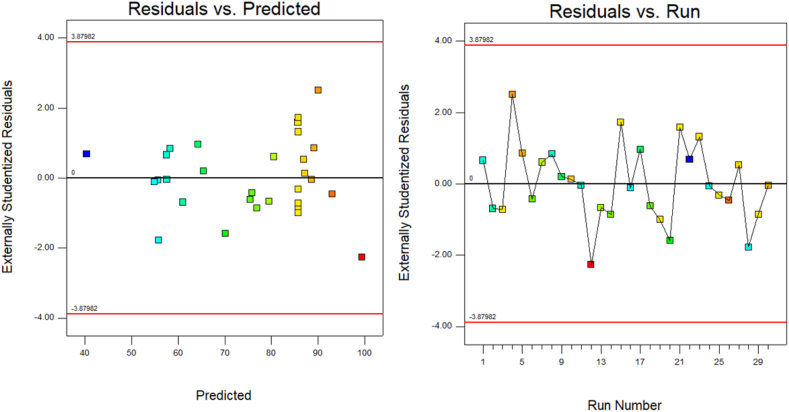
The diagnostic plots illustrate the analysis for detecting crystal violet using the proposed method. (For interpretation of the references to colour in this figure legend, the reader is referred to the Web version of this article.)

To assess the adequacy of the model, data from ANOVA (Table 3) was used. This statistical analysis helps evaluate the validity and significance of the proposed model. The quadratic model was selected, as it exhibited the lowest total sum of prediction error squares (1.66), an F-value of 16,961.36, a lack-of-fit F-value of 0.85, an adjusted R^2^ value of 0.9999, and a coefficient of variation of 0.22 %. The quadratic model confirms the adequacy of the model for determining crystal violet concentrations (Fig. 2).

**Table 3:** ANOVA for detecting crystal violet using the proposed method.

Source	Sum of squares	Degrees of freedom	Mean square	F-value	p-value	
Model	6309.95	14	450.71	16961.36	<0.0001	significant

A-P	1233.10	1	1233.10	46404.51	<0.0001	
B-S	982.40	1	982.40	36970.17	<0.0001	
C-T	1756.34	1	1756.34	66095.52	<0.0001	
D-V	1286.71	1	1286.71	48422.10	<0.0001	
AB	66.95	1	66.95	2519.62	<0.0001	
AC	0.32	1	0.32	11.91	0.0036	
AD	0.20	1	0.20	7.37	0.0160	
BC	0.26	1	0.26	9.69	0.0071	
BD	6.80	1	6.80	255.87	<0.0001	
CD	63.48	1	63.48	2388.95	<0.0001	
A^2^	86.29	1	86.29	3247.13	<0.0001	
B^2^	556.64	1	556.64	20947.67	<0.0001	
C^2^	280.63	1	280.63	10560.84	<0.0001	
D^2^	312.41	1	312.41	11756.74	<0.0001	
Residual	0.40	15	0.027			
Lack of Fit	0.25	10	0.025	0.85	0.6151	not significant
Pure Error	0.15	5	0.030			
Cor Total	6310.34	29

**Fig. 3:**
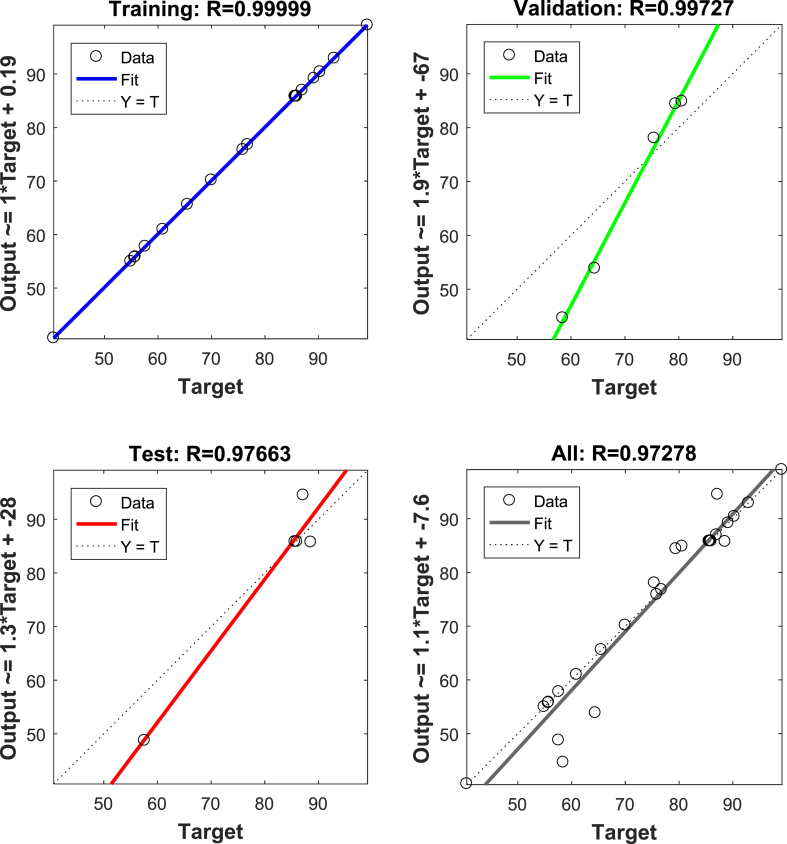
Correlation graphs between output and target values for detecting crystal violet. (For interpretation of the references to colour in this figure legend, the reader is referred to the Web version of this article.)

To assess the ANN model, the 30 runs were randomly divided into three sets. Table 2 displays the experimental design matrix for the ANN model for determining the amount of crystal violet. The aim was to achieve minimal MSE (mean square error) and maximal R^2^ values. The hidden layer was explored with 1–20 layers, and the optimized outcome was assayed to be 17 hidden layers. The input layer included parameters such as extraction solvent volumes, sodium chloride salinity, pH, and vortex time, while the output layer showed crystal violet extraction recovery. The outcomes demonstrated R^2^ values of 0.9999, 0.9972, and 0.9766 for training, validation, and test data, respectively, indicating high predictive accuracy of the network (Fig. 3). Similarly, the MSE values for the training, validation, and test sets were 0.0147, 70.1857, and 27.9873, which confirms the effectiveness of the ANN model.

The extraction recovery (ER) rate was used to assay crystal violet, which was defined according to Equation (1).

(1) $$\begin{array}{cc} \%\text{ER}= & \left(\frac{C_{2}}{C_{1}}\right)\times \left(\frac{V_{2}}{V_{1}}\right)\times 100 \end{array}$$

where C_1_ and C_2_ represent the initial concentration in the sample and sedimented phase. V_2_ and V_1_ represent the sedimented phase volume and aqueous solution volume.

The hydrophobic alkyl chain of the 1-octyl-3-methylimidazolium cation may interact with the hydrophobic regions of tetrahydrofuran molecules, leading to the formation of a supramolecular solvent. This supramolecular solvent can exhibit unique properties and is used in the extraction of crystal violet. The extraction of crystal violet dye into the supramolecular solvent is driven by a combination of electrostatic, hydrophobic, and π-π interactions, aided by the solvation properties of tetrahydrofuran. These mechanisms collectively ensure that the dye is effectively partitioned into the supramolecular solvent, allowing for efficient extraction from aqueous solutions.

**Fig. 4:**
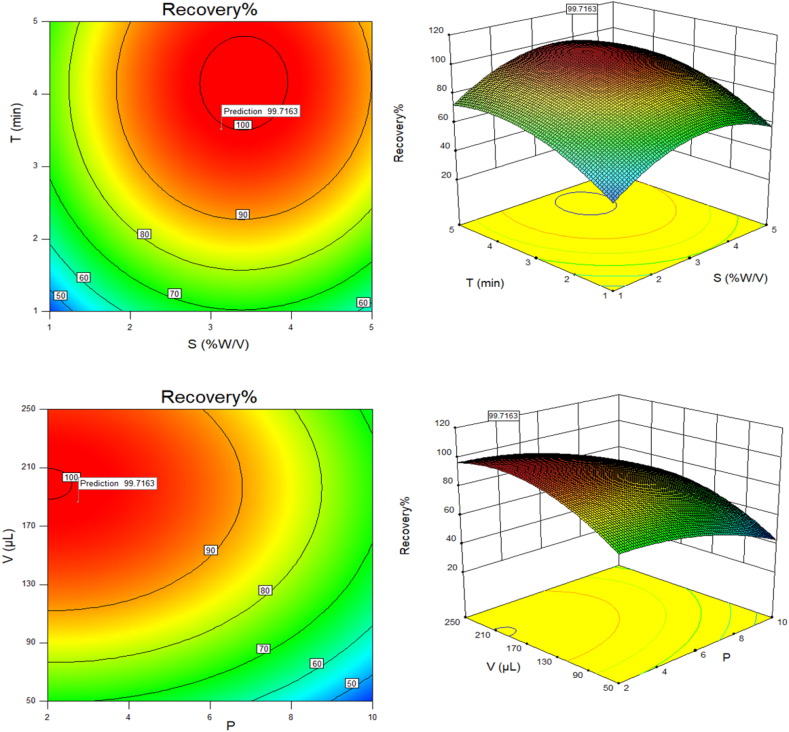
The surface plots for **P**: pH (2, 4, 6, 8, 10), **S**: Salinity, sodium chloride (1, 2, 3, 4, 5 %W/V), **T**: Time of extraction (1, 2, 3, 4, 5 min), **V**: Volume of solvent (50, 100, 150, 200, 250 μL) in detecting crystal violet. (For interpretation of the references to colour in this figure legend, the reader is referred to the Web version of this article.)

Fig. 4 demonstrates the combined effects of various parameters on the extraction process of crystal violet. The extraction efficiency increases with decreasing pH and increasing salt concentration or extraction time. According to Fig. 4, lower pH values are more favorable for the extraction of crystal violet, with the highest efficiency observed in the pH range of 2–4. Optimal extraction is achieved at a pH of 2.7 and a vortexing time of 3.5 min. Additionally, decreasing the solution pH and increasing the solvent volume enhances the extraction, with maximum efficiency at a solvent volume of 186 μL. Similarly, increasing the salt concentration while reducing vortex time or solvent volume also improves extraction efficiency. The optimal conditions identified using RSM for crystal violet extraction are a pH of 2.7, 186 μL of extraction solvent, a vortex time of 3.5 min, and a salt concentration of 3.1 % w/v. The validity of the proposed model was confirmed through crystal violet detection. The predicted results closely match the experimental observations, with a validation error of 1.07 %–2.45 %, which is within the acceptable limits according to the method's criteria. This highlights the model's effectiveness in accurately predicting the relationship between input and output variables in crystal violet detection.

### 3.2 Selectivity

To evaluate the selectivity of the micro-extraction method using a supramolecular solvent, the interference effect on crystal violet determination was studied. The extraction of crystal violet was tested in the presence of several interfering materials in solutions containing 200 ng mL⁻^1^ of crystal violet. Table 4 provides the threshold limits for specific interfering species, representing the maximum concentrations of these compounds that still permit accurate measurement without significantly affecting crystal violet recovery. A substance was considered an interfering factor if it caused more than a ±5 % change in the analytical signal. The results indicate that, even in the presence of varying concentrations of potential interferences, the micro-extraction method using supramolecular solvent consistently yielded satisfactory extraction results for crystal violet (Table 4).

**Table 4:** Effect of some ions and dyes on the recoveries of CV (n = 3).

Interfering substances	Tolerance ratio (mg L^−1^)	%Recovery± SD	Foreign species	Tolerance ratio (mg L^−1^)	%Recovery± SD
Cl^−^	500	98.92 ± 2.86	Na^+^	500	98.82 ± 2.86
Br^−^	500	100.21 ± 3.10	Ca^2+^	500	98.80 ± 2.96
F^−^	500	97.80 ± 2.73	Mg^2+^	500	98.32 ± 4.03
Ni^2+^	500	99.80 ± 2.89	Zn^2+^	300	102.03 ± 2.75
Co^2+^	300	98.71 ± 2.66	Pb^2+^	300	98.85 ± 4.05
Cu^2+^	300	98.36 ± 2.65	Cd^2+^	300	99.53 ± 2.88
Fe^2+^	100	98.85 ± 2.76	Fe^3+^	100	101.07 ± 2.93
Rhodamine B	50	99.76 ± 2.89	Methylene blue	50	99.41 ± 2.86
Malachite green	25	102.34 ± 3.88	Brilliant green	25	100.74 ± 3.82

### 3.3 Performance of the procedure

The figures of merit for the proposed method were determined under optimized values. The detection limit was 2 ng mL⁻^1^ (n = 10), with a calibration curve linear over the range 10–800 ng mL⁻^1^ (Abs = 0.0027C (ng mL⁻^1^) + 0.031, R^2^ = 0.9985). The percentage relative standard deviation (%RSD) was calculated to assess the repeatability (intra-day) and reproducibility (inter-day) of the procedure. The %RSD (n = 5, 200 ng mL⁻^1^ CV) was 2.7 % for repeatability and 3.9 % for reproducibility. The enrichment factor, calculated as the ratio of the slopes of calibration curves for pre-concentrated samples to those obtained without the pre-concentration method, was 90-fold. To assess the performance of suggested model in real samples, tests were conducted, with the standard addition method employed to account for potential matrix effects. As shown, the method achieved quantitative recovery values for crystal violet from complex samples, with recoveries ranging from 97.42 % to 99.88 % (Table 5). Therefore, we can confidently conclude that the suggested model is effective for detecting crystal violet in water samples.

**Table 5:** Application of the proposed model to determine CV (n = 3).

Samples	Added, ng mL^−1^	Found, ng mL^−1^ (mean ± SD)	%Recovery
Carp Fish	0.00	BDL	–
	40.00	39.6 ± 1.15	99.00
	120.00	118.7 ± 3.21	98.92
	250.00	245.4 ± 7.36	98.16
Fish farming water	0.00	BDL	–
	40.00	39.5 ± 0.98	98.75
	120.00	118.2 ± 3.42	98.50
	250.00	249.6 ± 6.24	99.84
River water	0.00	BDL	–
	40.00	39.8 ± 1.12	99.50
	120.00	116.9 ± 3.15	97.42
	250.00	249.7 ± 7.74	99.88

### 3.4 Compare method

A brief comparison of the proposed approach with several published methods for the extraction and determination of crystal violet was conducted (Table 6). As shown, our approach offers comparable or better performance to some of the referenced models, including easier experimental procedures, lower detection limit, lower cost, a simpler extraction process, and reduced solvent usage [41,42,44,45]. Despite the lower detection limit outlined in Refs. [40,43,46], these approaches utilize sensitive analytical devices, such as HPLC and MS, these instruments are expensive, making their widespread application for crystal violet detection in developing countries challenging. Overall, the proposed approach was optimized using a systematic testing technique for enhancing extraction recovery of crystal violet while preserving the simplicity, sensitivity, and reliability. The accessibility, cost-effectiveness of the equipment, simplicity, efficiency, and low detection limit highlight the usefulness of the suggested model for the determination of crystal violet.

**Table 6:** Comparison of the proposed model with the reported methods for CV.

Method	Recovery (%)	Linear range (ng mL^−1^)	LOD (ng mL^−1^)	RSD%	Ref
PDMS-PDA-SBE-MS	88.6–101.2	0.1–100	0.16	5.9	[40]
GO-DSPE-PLS-UV-Vis	90.78–101.61	30–300	9	1.99–0.58	[41]
PMD-μ-SPE-UV-Vis	97.4–111.3	50–7000	50	4.6–7.9	[42]
SA-GO-DSPME-HPLC	93.10–113.90	1–200	0.3	3.8	[43]
SPE-PLS	76.4–90.6	120–8000	28	1.8–7.3	[44]
CE-UV	89.1–99.0	980–100000	290	0.9–3.0	[45]
ELLME-DES-HPLC	79.63–92.90	0.2–200	0.13	2.3	[46]
SUPRAS-VA-DLLME-UV-Vis	97.42–99.88	10–800	2	2.7	This work

PDMS-PDA-SBE-MS: Polydimethylsiloxane-polydopamine-stir bar extraction - mass spectrometry, GO-DSPE-PLS: Graphene oxide-dispersive solid phase extraction-partial least squares, PMD-μ-SPE: Polypyrrole-magnetite dispersive micro solid phase extraction, SA-GO-DSPME: Salt-assisted graphene oxide dispersive solid phase micro-extraction, SPE-PLS: solid phase extraction-partial least squares, CE-UV: Capillary electrophoresis, ELLME-DES: Emulsifcation liquid–liquid micro-extraction-deep eutectic solvent, SUPRAS-VA-DLLME: Supramolecular solvent-vortex assisted-dispersive liquid-liquid microextraction.

## 4 Conclusion

This study investigated the feasibility of determining crystal violet in water samples using micro-extraction based on the supramolecular solvent phase. Through systematic experimentation, critical process parameters were optimized to enhance the efficiency of crystal violet extraction. The optimal conditions identified were a pH of 2.7, 186 μL of extraction solvent, a vortex time of 3.5 min, and a salt concentration of 3.1 % w/v. A quadratic model was selected, as it demonstrated the lowest total sum of prediction error squares (1.66), an F-value of 16,961.36, a lack-of-fit F-value of 0.85, an adjusted R^2^ of 0.9999, and a coefficient of variation of 0.22 %. The ANN model, configured as 4-17-1, also performed exceptionally well, with MSE and R^2^ data for training (0.0147, 0.9999), validation (70.1857, 0.9972), and testing (27.9873, 0.9766), confirming the effectiveness of the proposed ANN. Ultimately, the developed procedure successfully determined crystal violet in real samples, achieving satisfactory extraction recovery rates ranging from 97.42 % to 99.88 %.

## CRediT authorship contribution statement

**Najmeh Sanjarani:** Writing – original draft, Resources, Data curation, Conceptualization. **Mashaallah Rahmani:** Writing – review & editing, Validation, Supervision, Project administration, Methodology.

## Declaration of competing interest

The authors declare that they have no known competing financial interests or personal relationships that could have appeared to influence the work reported in this paper.
